# Neurosyphilis: Old Disease, New Implications for Emergency Physicians

**DOI:** 10.5811/cpcem.2019.9.43871

**Published:** 2019-11-19

**Authors:** Laura Mercurio, Lynn E. Taylor, Angela F. Jarman

**Affiliations:** * Alpert Medical School of Brown University, Department of Emergency Medicine, Providence, Rhode Island; † University of Rhode Island Providence Campus, CODAC Behavioral Health, Providence, Rhode Island; ‡ Alpert Medical School of Brown University, Department of Pediatrics, Providence, Rhode Island

## Abstract

Recent epidemiologic data demonstrate increasing rates of neurosyphilis, particularly among those in the community of men who have sex with men and those coinfected with the human immunodeficiency virus (HIV). Here we discuss a case of early neurosyphilis and new HIV diagnosis in a 27-year-old previously-healthy trans woman presenting for the second time with progressive, ascending weakness and cranial nerve VI palsy. Emergency physicians should consider this rare but highly morbid diagnosis, given the rising prevalence of neurosyphilis among at-risk patients and those with new neurologic deficits.

## INTRODUCTION

Neurosyphilis, an invasion of the central nervous system (CNS) by the spirochete *Treponema pallidum (T pallidum)*, can affect the brain parenchyma, meninges, and spinal cord. This disease has been commonly understood as the consequence of chronic, untreated syphilis. Now early neurosyphilis is resurging among at-risk populations in the United States (U.S.) and abroad. During 2015–2016, the U.S. national syphilis rate increased by 17.6% to 8.7 cases per 100,000 population – the highest rate reported since 1993.^1^ The ratio of biologically male to female individuals with syphilis also increased from 1.2 (1996) to 5.7 (2005) per 100,000^2^; this increase was disproportionately seen among those in the community of men who have sex with men.^3^

By some estimates, up to 75% of new syphilis cases were among the men who have sex with men population, and approximately half of these individuals were coinfected with human immunodeficiency virus (HIV).^4^,^5^ The prevalence of neurosyphilis among HIV-infected individuals with untreated syphilis has been reported as high as 23.5–40%, as compared to approximately 10% in HIV-uninfected individuals.^6^ Here we present a case of neurosyphilis in a reportedly previously-healthy trans woman presenting with progressive, ascending weakness and new HIV diagnosis.

## CASE REPORT

A 27-year-old male-to-female transgender patient with no significant medical history presented to the emergency department (ED) with three weeks of worsening lower extremity weakness and pain that progressed to include bilateral upper extremity weakness. She described her left leg “giving way” while at work several weeks prior; this episode caused a fall, but she was able to continue working and did not seek medical attention at that time. Within a week, her lower extremity weakness progressed to the point of requiring assistance to ambulate to the bathroom. She also developed worsening, diffuse left leg and low back pain.

The patient initially sought care one week prior to presentation at an outside hospital with the chief complaint of back and leg pain and weakness; she reported that radiographs of the lumbar spine were normal, and she was discharged home with ibuprofen and muscle relaxants. The patient subsequently developed worsening upper extremity weakness such that she could not pull herself up in bed. Review of systems indicated a 10-pound weight loss and intermittent double vision with rightward gaze; she denied any rash or lesions, fevers, chills, photophobia, headache, neck pain or stiffness, or any recent illnesses or vaccinations. The patient endorsed being sexually active with one male partner and did not use barrier protection during anal intercourse. She denied having prior HIV testing. Social history was notable for daily cigarette use, but no current or past intravenous (IV) drug use. The patient had no family history of multiple sclerosis, amyotrophic lateral sclerosis, or other neurodegenerative diseases.

The patient had normal vital signs on presentation. Initial neurologic examination was notable for an alert, anxious, cooperative patient with fluent speech, loss of right eye abduction with diplopia, leftward tongue deviation, 3/5 strength in right lower extremity, 1/5 strength in left lower extremity, 4/5 strength of bilateral upper extremities, 1+ biceps reflexes with absent patellar reflexes bilaterally. She was unable to walk due to weakness, and required some assistance to move from lying to sitting in bed.

Pertinent findings on serum testing included the following: a normal c-reactive protein of 2.98 milligrams per deciliter (mg/dL) (reference [ref] 0.0–10.0 mg/dL), an elevated erythrocyte sedimentation rate of 123 millimeters per hour (mm/hr) (ref 0.0–20.0 mm/hr), a normal white blood cell count of 6.3 ×10^9^/liter (L) (ref 4.4–16.0 ×10^9^/L) with a slight neutrophilic predominance, but without bandemia (67.9% neutrophils, 19% lymphocytes 11.2% monocytes 1.4 eosinophils, 0.5 bands); and an elevated creatinine kinase (336 international units [IU]/L, ref 20–210 IU/L). Other studies that were within normal limits included hemoglobin (13.7 grams [G]/dL, ref 11.0–16.3 G/dL); platelet count (231 ×10^9^/L, ref 150–400 ×10^9^/L); basic metabolic panel; liver function testing; and thyroid stimulating hormone.

A non-contrast computed tomography of the brain was obtained and was notable for a 1.5 centimeter-mass within the central left nasopharynx but did not show any acute cerebral/cerebellar findings. Lumbar puncture was performed and cerebrospinal fluid (CSF) results included the following: normal glucose (56 mg/dL, ref 38–85 mg/dL); elevated protein (792 mg/dL, ref 15–45 mg/dL); and pleiocytosis (94 cells, ref 0–5/cubic millimeters) with 92% lymphocytes (ref 63–99%). Ceftriaxone and vancomycin were initiated based on abnormal CSF preliminary testing.

Given the significant weight loss and new neurologic deficits, we obtained consent for HIV testing, and rapid antibody/antigen testing returned positive. A qualitative serum rapid plasma reagin (RPR) was sent and found to be positive with a titer of 1:16. CSF herpes simplex virus I/II polymerase chain reactions were negative. While in the ED, CSF venereal disease research laboratory (VDRL) testing was positive with a 1:2 titer, at which point 24 million units of IV penicillin G was administered. HIV myositis was also considered, although creatinine kinase level was only 316 IU/L (ref 20–210 IU/L). After a multidisciplinary discussion with neurology and immunology, neither steroids nor IV immunoglobulin (IG) were administered in the ED pending further imaging and serologic evaluation.

**CPC-EM Capsule**

What do we already know about this clinical entity?

*Neurosyphilis is an invasion of the central nervous system by the spirochete* Treponema pallidum*. This disease has been understood as a consequence of chronic, untreated disease, but can present as a primary infection.*

What makes this presentation of disease reportable?

This case demonstrates a case of primary neurosyphilis with HIV diagnosis in a previously-healthy trans woman with progressive lower-extremity weakness.

What is the major learning point?

It is important to consider neurosyphilis among HIV-infected patients, as well as those within the men who have sex with men and transgender communities.

How might this improve emergency medicine practice?

Practitioners should consider neurosyphilis in at-risk patients, those with HIV or new, unexplained neuro-psychiatric symptoms.

Magnetic resonance imaging with and without contrast of the brain and spine was notable only for diffuse enhancement of cauda equina nerve roots with minimal, associated nerve root thickening, but no definite nodularity or clumping (Image).

The patient completed a 14-day course of IV penicillin G therapy (24 million units) for neurosyphilis. The initial cluster of differentiation 4 (CD4) count was 0.247 K/uL (ref 0.500–1.800 K/uL) with HIV RNA vial load of 37,811 copies/mL (ref 20–10,000,000 copies/mL). She was started on a once-daily antiretroviral regimen of elvitegravir, cobicistat, emtricitabine, and tenofovir alafenamide after resistance testing was sent. She also received five days of IVIG due to concern for Guillain-Barré syndrome (GBS). It was ultimately unable to be determined whether her weakness stemmed from neurosyphilis alone, GBS, HIV-related neuropathy, critical illness myopathy, or a combination of these. Blood cultures and CSF cultures remained negative. Cryptococcal, human T-lymphotrophic virus 1, and herpes simplex virus testing were negative.

After hospital discharge, the patient required 15 days of inpatient physical rehabilitation, but ultimately signed out against medical advice. She reported improvement in diplopia but continued to have lower extremity weakness requiring a walker for mobility and assistance with activities of daily living. A three-month follow-up lumbar puncture was planned to reassess CSF pleiocytosis and VDRL testing, but was not completed. Bloodwork obtained 10 months after initial diagnosis included reactive RPR with a titer of 1:4; negative hepatitis B panel; negative mycobacterium tuberculosis testing; and an undetectable HIV RNA viral load. Currently, the patient remains lost to in-person follow-up due to ambulation and transportation difficulties.

## DISCUSSION

Progressive, lower extremity weakness has a wide differential diagnosis ranging from isolated neuropathies, spinal cord compression syndromes, and inflammatory or autoimmune disease (e.g., multiple sclerosis, GBS), to CNS infection and oncologic processes. This case highlights the importance of considering neurosyphilis among HIV-infected patients, as well as those within the men who have sex with men and transgender communities.

Estimates report 20–50% of men who have sex with men with syphilis living in major cities have concurrent HIV infection.^2^ Prior or concurrent HIV infection increases risk of CNS invasion in early syphilis – a 2004 study demonstrated that 2.1% of HIV-infected individuals presented with neurosyphilis as their early disease manifestation, while only 0.6% of HIV-uninfected individuals presented in this manner.^7^ The four sub-types of neurosyphilis (Figure) are based on natural history of syphilis infection: symptomatic meningitis; meningovascular disease; general paresis; and tabes dorsalis. CNS infection can present with a broad range of neurologic concerns including psychiatric symptoms, dementia, headache, stroke-like symptoms, meningismus, progressive weakness, and encephalopathy.^4^,^8^–^10^

The diagnostic approach to suspected neurosyphilis includes lumbar puncture and serum studies to test for the presence of *T pallidum* in the blood and CSF.^4^,^10^ Syphilis diagnostic testing can be divided into two categories: non-treponemal testing – RPR and VDRL; and treponemal testing – most commonly, the fluorescent treponemal antibody absorption (FTA-ABS) test. Non-treponemal testing detects the presence of antibody/antigen to *T pallidum* proteins, while the FTA-ABS tests for the whole organism.^4^ Nontreponemal tests can be falsely non-reactive in late syphilis, while treponemal tests remain reactive for life in patients with all forms of syphilis, even after treatment.^4^

Initial evaluation typically includes serum FTA-ABS and CSF VDRL testing (qualitative, then quantitative), as well as CSF culture and cell counts. *T pallidum* antibodies (immunoglobulin-G, immunoglobulin-M) can also be obtained to evaluate chronicity of syphilis infection.^11^ Since this case, revised U.S. Centers for Disease Control and Prevention guidelines recommend starting with treponemal testing.^12^ HIV testing should also be performed if the patient’s status is not already established. HIV-infected patients with neurosyphilis can present with unique clinical manifestations including initial false negative serologic testing due to the prozone phenomenon; rapid progression to meningovascular disease; and inconsistent CSF leukocytosis. However, CSF testing should reveal an elevated protein, and this population is more likely to have a Jarisch–Herxheimer reaction – a systemic inflammatory response to treatment caused by the rapid lysis of treponemal organisms. Individuals with a new HIV diagnosis should also undergo appropriate CSF testing for opportunistic infections including tuberculosis, herpes simplex viruses and, depending on clinical presentation, human polyoma virus 2.

Standard treatment for neurosyphilis includes 18–24 million units/day of IV penicillin G therapy for 10–14 days.^6^ Our patient required a 14-day course of penicillin as well as antiretroviral therapy. Repeat CSF testing should be performed approximately three months^13^ after treatment to confirm clearance of the *T pallidum* infection from the CNS. Of note, HIV-infected individuals may fail to clear the anti-treponemal antibodies after therapy, but there should be a significant decrease in levels as measured by CSF VDRL and plasma RPR testing.^6^,^10^ Patients also often report persistent neurologic symptoms six months after initial diagnosis and treatment.^14^

## CONCLUSION

Given the rising prevalence of neurosyphilis, emergency physicians should consider this “cannot-miss” diagnosis, particularly among patients presenting with progressive weakness, unexplained psychiatric symptoms, new-onset dementia, or focal neurologic findings. These patients should also receive HIV testing given the rate of co-infection, unique clinical manifestations, and response to treatment.

## Figures and Tables

**Image:**
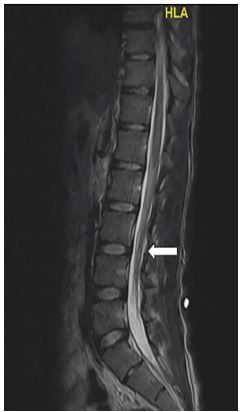
T2-weighted magnetic resonance imaging of the spine. Lower spinal sagittal windows demonstrate diffuse enhancement of the cauda equina (white arrow).

**Figure:**
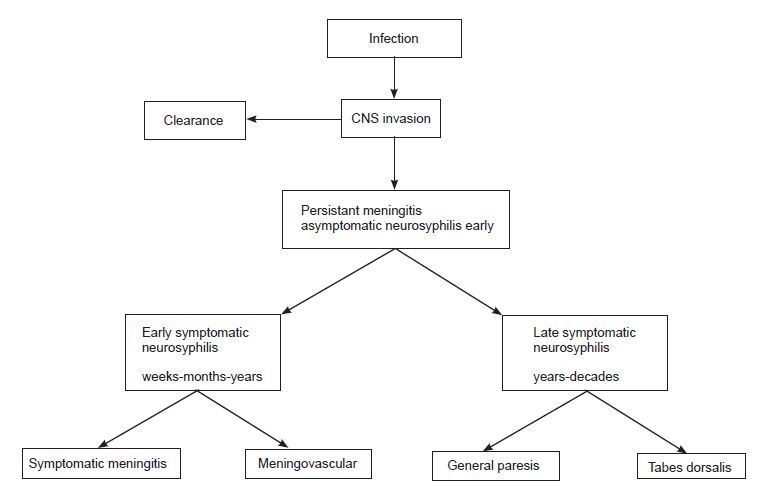
Natural history of neurosyphilis. Neuro-invasion occurs in at least 40% of individuals. Clearance occurs in about 70% of individuals. The remaining 30% of patients have persistent central nervous system (CNS) infection, also called asymptomatic neurosyphilis. In the pre-penicillin era, about 20% of individuals with asymptomatic neurosyphilis developed one of the symptomatic forms of neurosyphilis. In the penicillin era, the early forms (eg, symptomatic meningitis, meningovasculitis) are more common than the late forms (e.g., dementia and tabes dorsalis). Reprinted with permission from Marra CM.^4^
